# Boron deficiency in woody plants: various responses and tolerance mechanisms

**DOI:** 10.3389/fpls.2015.00916

**Published:** 2015-10-27

**Authors:** Nannan Wang, Chengquan Yang, Zhiyong Pan, Yongzhong Liu, Shu’ang Peng

**Affiliations:** Key Laboratory of Horticultural Plant Biology (Ministry of Education), Department of Pomology, College of Horticulture and Forestry Science, Huazhong Agricultural UniversityWuhan, China

**Keywords:** boron efficiency, boron reserves, cell wall, lignin, transporter, trees

## Abstract

Boron (B) is an essential microelement for higher plants, and its deficiency is widespread around the world and constrains the productivity of both agriculture and forestry. In the last two decades, numerous studies on model or herbaceous plants have contributed greatly to our understanding of the complex network of B-deficiency responses and mechanisms for tolerance. In woody plants, however, fewer studies have been conducted and they have not well been recently synthesized or related to the findings on model species on B transporters. Trees have a larger body size, longer lifespan and more B reserves than do herbaceous plants, indicating that woody species might undergo long-term or mild B deficiency more commonly and that regulation of B reserves helps trees cope with B deficiency. In addition, the highly heterozygous genetic background of tree species suggests that they may have more complex mechanisms of response and tolerance to B deficiency than do model plants. Boron-deficient trees usually exhibit two key visible symptoms: depression of growing points (root tip, bud, flower, and young leaf) and deformity of organs (root, shoot, leaf, and fruit). These symptoms may be ascribed to B functioning in the cell wall and membrane, and particularly to damage to vascular tissues and the suppression of both B and water transport. Boron deficiency also affects metabolic processes such as decreased leaf photosynthesis, and increased lignin and phenol content in trees. These negative effects will influence the quality and quantity of wood, fruit and other agricultural products. Boron efficiency probably originates from a combined effect of three processes: B uptake, B translocation and retranslocation, and B utilization. Root morphology and mycorrhiza can affect the B uptake efficiency of trees. During B translocation from the root to shoot, differences in B concentration between root cell sap and xylem exudate, as well as water use efficiency, may play key roles in tolerance to B deficiency. In addition, B retranslocation efficiency primarily depends on the extent of xylem-to-phloem transfer and the variety and amount of *cis*-diol moieties in the phloem. The B requirement for cell wall construction also contribute to the B use efficiency in trees. The present review will provide an update on the physiological and molecular responses and tolerance mechanisms to B deficiency in woody plants. Emphasis is placed on the roles of B reserves that are more important for tolerance to B deficiency in trees than in herbaceous plants and the possible physiological and molecular mechanisms of differential B efficiency in trees. We propose that B may be used to study the relationship between the cell wall and the membrane via the B-bridge. Transgenic B-efficient tree cultivars have considerable potential for forestry or fruit rootstock production on low B soils in the future.

## Introduction

Boron (B) is essential for the growth and development of higher plants (Warington, 1923). In the past 90 years, numerous significant advances have been made in understanding the function of B in plants. The current understanding of the biological function of B in plants is that it plays a structural role in both the cell wall and plasma membrane. In the cell wall, B forms borate esters with apiose residues of the pectic domain rhamnogalacturonan II (RG-II; Kobayashi et al., 1996). This B–RG-II complex is essential for cell wall structure and function (O’Neill et al., 2004) because it contributes significantly to the control of cell wall porosity (Fleischer et al., 1999) and tensile strength (Ryden et al., 2003). A recent study reported that glycosylinositol phosphorylceramides (GIPCs), the major components of lipid rafts, participate in the formation of the GIPC–B–RG-II complex, suggesting the structural role of B in membranes (Voxeur and Fry, 2014).

Boron deficiency is a widespread problem in both agriculture and forestry, particularly on sandy and alkaline soils (Bell and Dell, 2008). Areas where low B soils are found include South and Southeast Asia, Eastern Australia and New Zealand, Africa, North and South America, and Northern Europe (reviewed by Lehto et al., 2010). Low B causes significant losses of yield or quality by influencing vegetative or reproductive growth in forest trees (Stone et al., 1982; White and Krause, 2001; Lehto et al., 2010), fruit trees (Raja et al., 2005; Kumar, 2011; Ganie et al., 2013; Liu et al., 2013b) and woody plants (Broschat, 2005; Tewari et al., 2010; Hajiboland et al., 2011a; Patnude and Nelson, 2012). However, compared to model or herbaceous plants, in woody trees the understanding of B-deficiency responses and tolerance mechanisms is limited. Recently, advances have been made about the regulation of B transport both in herbaceous and woody plants. For instance, B transport system and its transporters were better understood (Miwa et al., 2013; Tanaka et al., 2013; Chatterjee et al., 2014; Durbak et al., 2014; Hanaoka et al., 2014); and studies of the signal transduction of B starvation responses were initiated (González-Fontes et al., 2013; Quiles-Pando et al., 2013; González-Fontes et al., 2014). These new findings provide important insights for understanding B deficiency in woody plants. Here, the recent achievements regarding the response and tolerance mechanisms of B deficiency in woody plants, as well as relevant findings in model or herbaceous plants, are summarized, and perspectives are also proposed.

## Physiological Responses To B Deficiency In Woody Plants

### Plant Growth and Visual Symptoms

Boron-deficient trees exhibit various visible symptoms in both vegetative and reproductive organs. Boron deprivation initially reduces the elongation of growing points due to restricted cell wall deposition and then, in more extreme cases, induces the necrosis of these tissues owing to cell death. This negative effect directly reduces root growth, particularly the lateral roots (Mei et al., 2011; Zhou et al., 2014). Boron deficiency also supresses growth in the aerial parts, such as plant height and leaf area (Möttönen et al., 2001; Wojcik et al., 2008). If B deficiency lasts for many years, it results in the stunted appearance of trees. In some forest trees, long-term B deficiency can reduce the quality and utility of wood. Loblolly pine (*Pinus taeda*), for example, can grow normally for the first 3 years and then experience dieback under low B conditions (Vail et al., 1961). Similar symptoms of dieback are also observed in other tree species (**Table 1**). Deficient symptoms in leaves, including chlorosis, necrosis or malformation, have been reported in many tree and woody species (**Table 1**). When grape (*Vitis vinifera*) was cultured under low B conditions, diffuse yellowing of the young leaves, brownish areas of apical tendrils and cupping of the third–fourth leaves from the shoot tips were observed in the early stage (cultured for 1 month). With the increasing time, the leaves became more cupped and chlorotic, and the tendrils developed transverse cracks and necrosis (Scott and Schrader, 1947). Mulberry (*Morus alba*), whose leaves are used to feed silkworm, changed to cup-shaped leaves with bent and cracked veins under B limitation (Tewari et al., 2010). In addition, reproductive growth, especially flowering, fruit set and yield, is more sensitive to low B than is vegetative growth (Dell and Huang, 1997). In grape, flower and fruit cluster necrosis, and small “shot berries” that are round to pumpkin-shaped often appear due to B starvation (Christensen et al., 2006). The papaya (*Carica papaya*) fruit is often affected by B deficiency with latex secretion and deformity (Wang and Ko, 1975). Usually, milky latex secretion appears in the fruit surface at the early stage, after which the milky latex becomes brown. Finally, the fruit surface becomes rough and deformed (Wang and Ko, 1975).

**Table 1:** Symptoms of B deficiency in trees.

Trees	Species	Symptom	Reference
Pine	*Pinus radiata*	Necrotic symptoms at the growing points	Ludbrook, 1940; Vail et al., 1961; Stone et al., 1982
	*Pinus taeda*	Needle fusion	
	*Pinus patula*		
	*Pinus elliotti*		
Spruce	*Picea abies*	Needle loss and top dieback	White and Krause, 2001; Lehto et al., 2010
	*Picea mariana*	Failure of budburst	
Eucalyptus	*Eucalyptus grandis*	Tissue dieback	Cooling and Jones, 1970
Leguminosae	*Dalbergia odorifera*	Leaf chlorosis	Lehto et al., 2010
	*Acacia mearnsii*	Top dieback	
	*Acacia mangium*		
Myrtaceae	*Eucalyptus urophylla*	Loss of apical dominance	Lehto et al., 2010
	*Eucalyptus globulus*	Prostrate plant	
Apple	*Malus pumila*	Internal or external lesions in the fruit Dieback and rosette in the vegetative parts	Burrell, 1940
Grape	*Vitis vinifera*	Chlorosis leaf and cupped young leaf Brownish areas developing in the apical tendrils Necrotic leaf and diebacked young tendrils Small “shot berries” Flower and fruit cluster necrosis	Scott and Schrader, 1947; Christensen et al., 2006
Papaya	*Carica papaya*	Deformed-fruit with “bumpy” skin surfaces	Wang and Ko, 1975; Nishina, 1991
Mango	*Mangifera indica*	Apical growing point and buds died Poor fruit set Internal necrosis in fruit Fruit cracking	Agarwala et al., 1988; Raja et al., 2005; Kumar, 2011
Blueberry	*Vaccinium corymbosum*	Tip dieback	Blevins et al., 1996
Almond	*Prunus dulcis*	Reduced pollen germination and tube growth	Nyomora et al., 2000
Citrus	*Citrus sinensis*	Pucker leaf with corky split veins	Han et al., 2008; Liu et al., 2013b; Yang et al., 2013; Zhou et al., 2014
	*Poncirus trifoliata*	Shoot tip necrosis	
	*Citrus sinensis ×*	Inhibition of plant growth	
	*Poncirus trifoliata*	Asymmetrical and deformed fruit	
	*Citrus reticulata*		
	*Citrus reshni*		
	*Citrus junos*		
	*Citrus aurantium*		
	*Citrus grandis*		
Cocoa	*Cacao theobroma*	Low yields and reduced fruit Malformed leaves, branches and fruits	Asomaning and Kwakwa, 1965; Tollenaar, 1967; Ojeniyi et al., 1981
Mulberry	*Morus alba*	Cup-shaped leaves Bent and cracked veins Lenticel-like cracks in petiole and stem	Tewari et al., 2010
Avocado	*Persea americana*	Reduced pollen viability and fruit size	Smith et al., 1997a,b
Tea	*Camellia sinensis*	Curling of leaf lamina Poorly branched root	Hajiboland et al., 2011b
Palm	*Cocos nucifera*	Necrotic truncation in an inverted “V” shape Multiple unopened spear leaves	Jayasekara and Loganathan, 1988; Broschat, 2005; Patnude and Nelson, 2012
	*Phoenix roebelenii*	Tiny crumpled leaves	
	*Bismarckia nobilis*	Severe epinasty	
	*Adonidia merrillii*	Premature fruit drop	
	*Roystonea regia*		
	*Syagrus romanzoffiana*		
	*Heterospathe elata*		

Taken together, B deficiency symptoms in trees can be divided into two main groups. One is the inhibition, even necrosis, of growing points, such as the root tip, bud, flower, and young leaf. Light microscopy observation showed that cell death occurred in B-deficient Norway spruce (*Picea abies*) needle buds (Sutinen et al., 2006, 2007), probably due to the B function in the cell wall. The other type of symptom is the deformity of some organs, such as the shoot, leaf, and fruit. Relevant anatomical studies demonstrated that B deficiency could severely damage the vascular tissues and induce hypertrophy at the tissue/cellular level. A disorganized vascular tissue was induced by B deficiency in coffee (*Coffea arabica*), and discontinuities of both xylem and phloem vessels were observed in the B-deficient stem tip and young leaf (Rosolem and Leite, 2007). Boron deficiency also reduced the length of the xylem vessel in both the leaf and fruit vascular bundles and reduced the diameter of the xylem vessel in only the leaf vascular bundle in pumelo (*Citrus grandis*; Liu et al., 2013b). A consistent observation reported is that B deficiency can increase vascular cross sectional areas in Norway spruce needle (Sutinen et al., 2006, 2007), pumelo leaf and fruit vascular tissues (Liu et al., 2013b), and sweet orange (*Citrus sinensis*) leaf veins (Yang et al., 2013). These results suggest that B deficiency can increase the size but weaken the function of vascular tissue in trees.

### Cell Wall and Membrane

Boron plays a crucial role in cell wall structure (O’Neill et al., 1996; Matoh, 1997; Brown et al., 2002; Goldbach and Wimmer, 2007). In B-deficient plants, the structures of the cell wall are strongly altered at both the macroscopic and microscopic levels (Loomis and Durst, 1992; Shorrocks, 1997). At the subcellular level, B starvation usually results in abnormally formed walls that are often thick and brittle as a consequence of altered mechanical properties and abnormal expansion (Brown et al., 2002). It has been suggested that B may be necessary for cell-to-wall adhesion and for the organization of the architectural integrity of the cell (Lord and Mollet, 2002; Bassil et al., 2004). This is further supported by an altered cell wall porosity and tensile strength under B deficiency (Fleischer et al., 1999; Ryden et al., 2003). In citrus and tea trees, B deprivation not only resulted in a heavily thickened and folded cell walls in roots (Zhou et al., 2015) but also increased the portion of the cell wall relative to the whole cell (Hajiboland et al., 2013b; Liu et al., 2013a) and induced changes in both the amount and assembly of its component polymers in leaves (Liu et al., 2014a). In forest trees, B deficiency impaired the primary cell wall, and interrupted the structural development of organs and whole plants, resulting in adverse impacts on tree formation, wood quality and cold tolerance (Lehto et al., 2010).

In general, the responses of membranes to B deficiency are faster than those of the cell wall (Goldbach et al., 2001; Brown et al., 2002). For example, within minutes of B deprivation, inhibition of plasma membrane-bound oxidoreductase activity was frequently observed (Barr et al., 1993). In addition, B-deficient plants often exhibit lower ion uptake rates in roots but higher eﬄux of potassium and organic compounds in leaves than B-sufficient plants (Pollard et al., 1977; Cakmak et al., 1995; Goldbach and Wimmer, 2007). The membrane-bound ATPase activity was reduced by B deficiency, but within 1 h after resupplying B, the activity was restored to the same level as that in B-sufficient bean (*Phaseolus vulgaris*) and maize (*Zea mays*) roots (Pollard et al., 1977). These results suggest that B deficiency might disturb the membrane transport process, the activity of membrane-located proteins, and the integrity and functioning of the plasma membrane (Brown et al., 2002; Goldbach and Wimmer, 2007; Camacho-Cristóbal et al., 2008b).

There is increasing evidence that B may play structural roles in the cell wall to cell membrane interface (O’Neill et al., 2001; Brown et al., 2002; Goldbach and Wimmer, 2007; Voxeur and Fry, 2014). Bassil et al. (2004) proposed that B may function in transvacuolar cytoplasmic strands and cell-to-wall adhesion. Recently, these predictions were partly confirmed by Voxeur and Fry (2014). These authors found that B can bind both the RG-II of the cell wall and the GIPCs of the cell membrane, thus forming a GIPC–B–RG-II complex (Voxeur and Fry, 2014). As a result, B serves as a bridge to connect the cell wall and the plasma membrane, which opens a possible avenue to probe the relationship between the cell wall and the membrane via the B-bridge. The GIPC–B–RG-II complex may also explain, at least partly, why both the cell wall and membrane are influenced by B deficiency.

### Metabolism

Early detectable changes in B-deficient plants are considered to be reflected by the damage of the cell membrane (see Cell Wall and Membrane) or the disturbances of hormonal metabolism (Blevins and Lukaszewski, 1998; Martín-Rejano et al., 2011; Abreu et al., 2014; Camacho-Cristóbal et al., 2015), but the primary reaction remains unclear. However, the accumulation of phenols has repeatedly been observed in B-deficient plants (Cakmak and Römheld, 1997; Marschner, 2012). It is believed that the accumulation of phenolic compounds is an indirect long-term effect of B deficiency (Marschner, 2012). Boron starvation first damages the integrity of the cell wall and membrane, disrupting the phenol metabolism-related enzyme systems, such as phenylalanine-ammonium lyase (PAL; Cakmak et al., 1995; Brown et al., 2002), and thus results in the accumulation of phenols and related alterations of lignin synthesis from phenol alcohols (Pilbeam and Kirkby, 1983; Yang et al., 2013; Zhou et al., 2015). In tea and olive trees, significant accumulation of phenolic compounds has been detected in B-deficient leaves (Liakopoulos et al., 2005; Hajiboland et al., 2013a). The excessive accumulation of phenols probably leads to tissue necrosis. The poorly lignified branches of woody trees due to B deficiency may be unable to support the weight of leaves (Dell and Huang, 1997). Additionally, changes in phenol and lignin may also affect plant defense systems against herbivory and pathogens (Lehto et al., 2010).

There is no evidence that B plays a direct role in photosynthesis (Dell and Huang, 1997). However, B deficiency limits root growth and results in a weak vascular tissue, which may restrain water uptake and transport within the plant and further alter leaf function (e.g., the reduction of stomata number and abnormal shapes, reviewed by Wimmer and Eichert, 2013). Moreover, a wealth of information is available to suggest that B deficiency may indirectly affect photosynthesis by decreasing the photosynthetic area and altering the leaf constituents (Dell and Huang, 1997; Brown et al., 2002). As in herbaceous species (Dell and Huang, 1997), B deficiency of leaves in trees reduces the content of chlorophyll, CO_2_ assimilation and stomatal conductance, as well as the activities of photosynthetic enzymes and catalase, but enhances the production of reactive oxygen species (ROS) and intercellular CO_2_ concentration, thereby resulting in decreased photosynthetic capability (Han et al., 2008; Wojcik et al., 2008; Tewari et al., 2010). Moreover, the accumulation of soluble sugars in B-deficient leaves of trees may also produce feedback inhibition to net photosynthesis (Han et al., 2008; Ruuhola et al., 2011; Hajiboland et al., 2013b; Lu et al., 2014).

### B Reserves

Interestingly, in woody perennial trees, B reserves play a significant role in response to B deficiency, particularly during the spring growth flush (Tromp, 1983; Spiegel-Roy and Goldschmidt, 1996). Boron reserves, also known as B storage, are defined as those B-containing substances that are not used directly in functioning but are primarily stored in the apoplast and cytoplasm of the tree until required (Tromp, 1983; Dannel et al., 2002; Du et al., 2002; Lehto et al., 2010). In contrast to herbaceous plants, trees are perennial and have a large body; therefore, trees may store adequate B to cope with B deficiency at a later stage. Generally, three B forms exist in plants: free B (B in the apoplast), semi-bound B (B in the cytoplasm), and bound B (B in the cell wall; Du et al., 2002). Previous studies have suggested that the forms of B reserves may be limited to free B and semi-bound B because bound B for cell wall construction is not retranslocated (Dannel et al., 2002; Matoh and Ochiai, 2005; Lehto et al., 2010; Liu et al., 2011; Wang et al., 2014).

Apart from the stage of growth (e.g., flowering and fruiting) and field management (e.g., leaf pruning; Goh et al., 2007), the amount of stored B in the plant is one of the most important factors that influence the B requirement of woody plants. Effective B application for trees is frequently observed, even though there is apparently sufficient B in leaves and no B-deficient symptoms evident (Hanson, 1991; Nyomora et al., 1999; Perica et al., 2001b; Sánchez and Righetti, 2005; Wells et al., 2008; Moura et al., 2013). Furthermore, the B-fertilized Norway spruce seedlings can maintain a foliar B concentration greater than the deficiency limit for 2–13 years, with larger trees having longer-lasting effects (Kilpeläinen et al., 2013; Riikonen et al., 2013). In addition, temporary B deficiency may occur in trees due to insufficient B reserves for redistribution within the plant, or limited B retranslocation in phloem, or to particular environmental conditions (e.g., low soil water and vapor pressure deficit; Bell, 2000). Hence, B application to enhance B storage in perennial organs, may result in increased B retranslocation, and thus improve the fruit set, yield, and wood quality of trees.

The effects of B application in woody plants are influenced by fertilization methods (Nyomora et al., 1999; Boaretto et al., 2011) and for foliar applications by leaf surface characteristics (Perica et al., 2001b; Will et al., 2012). Unlike annual plants, trees are perennial and commonly suffer from long-term B deficiency. For long-term B-deficient trees, the leaf structure may be more severely influenced than the root anatomy (see Plant Growth and Visual Symptoms), despite the faster response to B deficiency in roots than in shoots (Dell and Huang, 1997). This is because the root will not transport B to the shoot until its own essential requirements are met, as indicated by the fact that B retranslocation increases with an increase in B stored in the reserve tissues (the terms “B translocation” and “B retranslocation” used here refer to the processes of B transport in the xylem and phloem respectively, see details in Section B Translocation and Retranslocation; Boaretto et al., 2008; Lehto et al., 2010). In a limited-B-retranlocation species, the recovery of labeled B fertilizer in fruits was higher from soil application (21%) than for foliar application (7%; Boaretto et al., 2011). Interestingly, similar results were reported in a high-B-retranlocation species (Wojcik et al., 2008). Therefore, when applying B to B-deficient trees, application via the root system is more effective than foliar application, regardless of B retranslocation in plant species.

It has been suggested that stored B in older plant parts can be retranslocated to new tissues via the phloem during periods of rapid growth, and the extent of B retranslocation depends primarily on the B status and sugar alcohols in trees (Lehto et al., 2010). In sweet orange, 20–35% of the B content in new parts was retranslocated from plant B reserves (Boaretto et al., 2008). However, it should be noted that the proportion of B retranslocated varies more substantially among plant species than that of other essential plants nutrients (Brown and Shelp, 1997). For example, only 3.2% of newly acquired B by leaves was retranslocated in orange trees (Boaretto et al., 2011), whereas more than 70–80% of newly acquired B by leaves could be exported to young tissues in apple (*Malus domestics*), pear (*Pyrus communis*), prune (*Prunus domestics*), and sweet cherry (*Prunus avium*; Picchioni et al., 1995). Using the B concentration gradient along the shoot axis, and foliar ^10^B labeling, it has been demonstrated that 26 out of 31 deciduous tree species retranslocated B, while 10 out of 19 evergreen tree species did not retranslocated B in the phloem (Brown and Hu, 1998; Lehto et al., 2004b; Konsaeng et al., 2005). Furthermore, Aphalo et al. (2002) pointed out that the capacity for B retranslocation in conifer forests may be related to natural selection to avoid B stress in leaves with a long lifespan. These results indicate that the extent of B retranslocation may be greater in deciduous trees than in evergreen trees, probably due to the form and location of B reserves for each group of tree species during natural selection. The evolutionary advantage of B retranslocation is significant. Deciduous trees must retranslocate B from annual tissues to perennial tissues in a timely manner during the fall because the retranslocated B (also known as B reserve) plays a critical role in the growth of new organs in the subsequent spring. In contrast, evergreen trees do not experience defoliation and thus have less necessity to retranslocate B to new organs than do deciduous trees. This suggests that trees possess different adaptive strategies to B deficiency depending on climatic conditions.

## Molecular Responses To B Deficiency In Woody Plants

### B Transporters

In recent years, increasing numbers of papers on the molecular identification of B transporters in plants have been reported. More importantly, many B transporters are B-deficiency induced and their functions will become essential under low B conditions. To date, two types of B transporters have been identified: BORs, which perform a B export role in plant cells, and major intrinsic proteins (MIPs), including some boric acid channels (Miwa and Fujiwara, 2010; Reid, 2014). Both types of B transporters contribute to B uptake by roots (Takano et al., 2006; Durbak et al., 2014; Hanaoka et al., 2014), xylem loading and B distribution (Takano et al., 2001; Nakagawa et al., 2007; Tanaka et al., 2008) and B utilization within the plant under B-limited conditions (Miwa et al., 2013).

*Arabidopsis thaliana* BOR1, the first identified B exporter, plays a key role in xylem loading and B distribution within shoots (Takano et al., 2005). *At*BOR1 is expressed in the columella, lateral root cap, epidermis, and endodermis in the root tip, and in the epidermis and endodermis in the elongation zone (Takano et al., 2010). *AtBOR1* mRNA accumulation was not strongly expressed, while BOR1-GFP fusion protein accumulation was elevated under a limited B supply (Takano et al., 2005). *At*BOR2 is an eﬄux-type B transporter that is localized to the plasma membrane and has been proposed to facilitate the effective cross linking of RG-II by B in the cell wall and root cell elongation. This protein is strongly expressed in the lateral root caps and epidermis of the elongation zones of roots and is essential for root cell elongation under low B conditions (Miwa et al., 2013). *Os*BOR1, one of four *At*BOR1-like proteins in rice (*Oryza sative*), is a plasma-membrane-localized eﬄux transporter of B and is required for the normal growth of rice plants under B limitation (Nakagawa et al., 2007). *Os*BOR4, another *At*BOR1-like protein in rice, is both highly and specifically expressed in pollen. With the same transporter type and subcellular location as those of *Os*BOR1, *Os*BOR4 is essential for normal pollen germination and/or tube elongation in reproductive processes (Tanaka et al., 2013). *Zm*RTE (ROTTEN EAR), an ortholog of *At*BOR1 in maize, is also a B eﬄux transporter and required for inflorescence development and fertility under B-limited conditions (Chatterjee et al., 2014). In addition to these basic studies in model plants, homologs of *At*BOR1 exist in tree species. Both *Vv*BOR1 (Pérez-Castro et al., 2012) and *Citrus macrophylla* BOR1 (Cañon et al., 2013) are plasma-membrane-localized eﬄux B transporters. *VvBOR1* is mainly expressed in the root but also in other tissues. The relative expression of this gene in root is 1.9 times higher than that in flowers. Boron-deficient grape vines display symptoms of shot berries (Christensen et al., 2006; Pérez-Castro et al., 2012). However, at the fruit setting stage, the transcript accumulation of *VvBOR1* in shot berries is significantly less than that in normal berries (Pérez-Castro et al., 2012). *VvBOR1* transcripts increase at anthesis and then gradually decrease until late development stages during berry development. *CmBOR1* is expressed in the leaves, stem and flowers and shows the greatest level in the roots. A significantly increased expression of *CmBOR1* is observed in shoots under B-deficiency conditions (Cañon et al., 2013).

The MIP superfamily in plants can be subdivided into five evolutionarily distinct sub-families, including nodulin-26-like intrinsic proteins (NIPs), plasma membrane intrinsic proteins (PIPs), small basic intrinsic proteins (SIPs), tonoplast intrinsic proteins (TIPs) and uncharacterized X intrinsic proteins (XIPs) (Ishibashi et al., 2011). Some MIP members are boric acid channels facilitating B influx into cells. In *Arabidopsis*, both NIP5;1 and NIP6;1 are localized in the plasma membrane, but their B transport functions are different: NIP5;1 is involved in the initial uptake process in root cells (Takano et al., 2006), while NIP6;1 may function in xylem-phloem B transfer into young growing tissues (Tanaka et al., 2008). Genetic research on *Os*NIP3;1 (Hanaoka et al., 2014) and *Zm*NIP3;1 (also called TASSEL-LESS1 protein, TLS1; Durbak et al., 2014) demonstrated that they function as boric acid channels and are required for vegetative and reproductive development. Three PIP members, *Zm*PIP1 (Dordas and Brown, 2001), *Hordeum vulgare* PIP1;3 and *Hv*PIP1;4 (Fitzpatrick and Reid, 2009), increase the sensitivity of single cells to B, but their functions in whole plants are unclear. In citrus, an *AtNIP5*-like gene of trifoliate orange (*Poncirus trifoliata*), *CiNIP5*, is expressed mainly in the roots of citrus seedlings, and its transcripts increase significantly under B deficiency (An et al., 2012; Zhou et al., 2015).

Of the previously mentioned studies on B transporters, the majority involve model plants, whereas few were on tree species. In fact, the mechanism of B uptake, transport and distribution in woody plants is more complicated than current knowledge on model plants. For example, mycorrhiza exist in most herbaceous and woody plants and play an assistant role in the plant B uptake process (Lehto et al., 2004b; Ruuhola and Lehto, 2014). Unfortunately, no work has been reported on the existence of B transporters in mycorrhiza. Such studies would form a useful adjunct to research on B transporters in plants. However, the conserved function of B transporters is observed across model and woody species. *Vv*BOR1, for example, restores the phenotype of *Arabidopsis bor1-3* mutants under B deprivation (Pérez-Castro et al., 2012). Based on this consistency, the above new findings in model herbaceous plants could also provide a better understanding of the B deficiency response in woody plants. Moreover, to better understand the B transport system, studies are needed to identify and characterize potential B transporters in different tissue of trees, especially under B-deficiency conditions.

### Cell Wall Related Genes

As described in Section “Cell Wall and Membrane,” B deficiency causes abnormally formed cell walls that are often thick and brittle (Brown et al., 2002). Generally, the cell wall thickening process requires two elements: polysaccharides (e.g., cellulose and xylans) and aromatic components (e.g., lignins; Goujon et al., 2003). It is therefore likely that B deficiency may affect the expression patterns of cell-wall-related genes: decreasing production of molecules that are related to cell wall elements synthesis and inhibiting molecules that are related to cell wall extensibility modification. When *Arabidopsis* roots are under short-term B deficiency, decreased transcript counts of cell wall modification-related genes are observed by transcriptomic analysis (Camacho-Cristóbal et al., 2008a). In *Citrus* species, B deficiency suppressed the expression of cell-wall-modifying enzyme genes in the roots (Zhou et al., 2015) but increased the expression of lignin biosynthesis pathway genes in both the roots (Zhou et al., 2015) and leaf veins (Yang et al., 2013). These results suggest that B deficiency affects the expression of cell-wall-related genes in both herbaceous and woody plants.

### Signaling Transduction

Signal transduction in B-deficient plants is becoming an increasingly interesting topic. Three hypotheses for the B deprivation signaling pathway that transmits the signal from the cell wall to the cytoplasm and nucleus are summarized in a recent review (González-Fontes et al., 2014): (i) the alteration in cell wall structure promotes signal transduction; (ii) the accumulation of ROS induces Ca^2+^ signaling and cell death; and (iii) a low intracellular B level activates transcription factors and target genes. The authors have proposed a major role of Ca^2+^ and Ca^2+^-related proteins in this pathway (González-Fontes et al., 2014). Subsequently, crucial evidence supporting a membrane structure role of B was provided (Voxeur and Fry, 2014). Biological membranes contain not only lipids and proteins but also particular domains (rafts). These rafts are enriched in sterol and sphingolipids and are depleted in unsaturated phospholipids (Mongrand et al., 2010). In recent years, the possible role of plant rafts as signal transduction platforms has been frequently proposed and well summarized (see more information in Mongrand et al., 2010, and Simon-Plas et al., 2011). Moreover, as major components of membrane rafts, GIPCs participate in the formation of a GIPC–B–RG-II complex (Voxeur and Fry, 2014). Here, we propose a hypothesis, based on the negative effect on growing root cells and on the faster responses of membranes than those of the cell wall to B deficiency, and on a B deprivation signaling pathway. The hypothesis is as follows: a lack of the GIPC–B–RG-II complex for cell wall and membrane construction in root meristem and elongation cells might trigger the signaling pathway via the activation of a potential membrane protein or protein complex, and then the induced cytoplasmic Ca^2+^ signal pathway activates the downstream transcription events. Although little genetic or molecular evidence supports this hypothesis, phosphoproteomic analysis and single protein function research in both herbaceous and woody plant species will shed light on this topic.

## Mechanisms For Tolerance To B Deficiency In Woody Plants

Boron efficiency as used here refers to the extent of variation in response to low B among genotypes within one species and among plant species (Rerkasem and Jamjod, 1997). Boron-efficient genotypes are those that are able to grow well in soils in which other genotypes are adversely affected by B deficiency, and the opposite is the case for B-inefficient genotypes (Graham, 1984; Rerkasem and Jamjod, 1997). As in herbaceous plants (Bellaloui and Brown, 1998; Stangoulis et al., 2001; Nachiangmai et al., 2004; Rerkasem and Jamjod, 2004; Zhang et al., 2014), differential B efficiencies have also been observed in a variety of trees (Rerkasem and Jamjod, 1997; Lehto et al., 2004b; Xiao et al., 2007; Mattiello et al., 2009; Sheng et al., 2009a,b). It is widely accepted that the wide range of B efficiency among genotypes is associated with B uptake rate (B uptake efficiency), B translocation and retranslocation (B transport efficiency), and B utilization within the plants (B use efficiency; Marschner, 2012).

### B Uptake

At adequate to high B supply, B uptake occurs via passive diffusion across the lipid bilayer, whereas at low B supply, B in the external medium is initially taken up into the root symplasm through a passive facilitated transport process (Dannel et al., 2002; Miwa and Fujiwara, 2010). There are genotype-related differences in B uptake efficiency among trees. For example, under B-deficiency conditions, the sweet orange scion grafted on Carrizo citrange (*Citrus sinensis* ×*Poncirus trifoliata*) had a higher newly acquired B concentration in leaves than those grafted on trifoliate orange, suggesting that the rootstock Carrizo citrange has a greater B uptake efficiency than trifoliate orange (Liu et al., 2012). Boron uptake efficiency of trees has been suggested to be associated with root morphology (Mei et al., 2011) and mycorrhizas (Lehto et al., 2004b; Ruuhola and Lehto, 2014). Under low B conditions, B-efficient tree cultivars usually show less depression of root length and number (Mei et al., 2011) and thereby exhibit a higher B absorption rate (Wojcik et al., 2003; Han et al., 2012) compared to B-inefficient cultivars. Mycorrhizas, which often exist in symbiosis with trees, may also play an important role in B uptake efficiency. In silver birch (*Betula pendula*), B uptake rate was higher in *Laccaria*-inoculated than *Laccaria*-non-inoculated seedlings (Ruuhola and Lehto, 2014). At the molecular level, the *AtNIP5;1* gene is a boric acid channel that is involved in the initial uptake process in root cells (Takano et al., 2006). The overexpressed lines for this gene have greater root elongation under B-limited conditions (Kato et al., 2009). These results suggest that greater accumulation of transcripts of the boric acid channel gene could increase tolerance to B starvation by enhancing the initial uptake process. This suggestion is further supported by the fact that the *CiNIP5* transcript level in the roots of B-efficient Carrizo citrange increased continuously to 7.7 times at 48 h after B-deficiency treatment compared to the initial level, whereas that of B-inefficient fragrant citrus (*Citrus junos*) increased only to 4.4 times at 24 h and then decreased (An et al., 2012). A similar observation was also recently described in that the level of *NIP5;1* mRNA in roots after 12 h of B deficiency was up-regulated 5.2 times for B-efficient Carrizo citrange, but only 3.8 times for B-inefficient trifoliate orange (Zhou et al., 2015).

### B Translocation and Retranslocation

At adequate to toxic B supply, a substantial retention of B in the root symplasm occurs at xylem loading; at low B supply, the B retained in the symplasm can be loaded into the xylem by an active transport system (Dannel et al., 2002). Once loaded into the xylem, B can be translocated from the root to shoot by transpiration under high B conditions, but by active transport processes at low B supply (Raven, 1980; Shelp et al., 1995; Eichert and Goldbach, 2010; Miwa and Fujiwara, 2010), both of which are influenced by water use efficiency (Wimmer and Eichert, 2013). In general, B translocation efficiency is evaluated by the ratio between B concentration in the root cell sap and xylem exudate using a stable isotope ^10^B tracer. Under B-deficiency conditions, B-efficient genotypes usually have relatively higher B concentrations in xylem exudates than B-inefficient genotypes, probably due to the greater ability to translocate B from the root to the shoot. For example, at low B supply, the B-efficient tomato (*Solanum lycopersicum*) cultivar ‘Rutgers’ had a higher B concentration in xylem exudate than the B-inefficient cultivar ‘T3238’, but a similar B concentration was found in the root cell sap of both genotypes (Brown and Jones, 1971). Furthermore, ‘Rutgers’ was more efficient than ‘T3238’ in translocating ^10^B from the root to the shoot (Bellaloui and Brown, 1998). For grafted trees, in addition to functioning in B uptake by roots, the rootstock may also play a role in B translocation from the root to the scion (Papadakis et al., 2003; Wojcik et al., 2003; Boaretto et al., 2008; Sheng et al., 2009b; Wang et al., 2014). Under B-deficiency conditions, the ratio of B concentration in the scion stem to the rootstock stem increased as the B efficiency of citrus combinations increased (Wang et al., 2014). This implies, at least in part, that B-efficient grafted combinations possess a greater ability to translocate B from the rootstock (root) to the scion (shoot). The B translocation efficiency in trees is likely related to water use efficiency in rootstocks and scions, and to connectivity in the grafting region, presumably due to the B transport in xylem associated with aquaporins (Wimmer and Eichert, 2013). Compared to the B-inefficient citrus rootstock trifoliate orange, the B-inefficient genotype Carrizo citrange is a vigorous rootstock that usually has a higher vessel diameter and hydraulic conductance (Saeed et al., 2010; Mei et al., 2011; Zhou et al., 2014). However, whether B translocation efficiency is associated with xylem vessel characteristics and water use efficiency still needs to be explored. At the molecular level, BOR1 proteins have been identified as B eﬄux transporters that are involved in B xylem translocation in the roots of *Arabidopsis* (Takano et al., 2001), rice (Nakagawa et al., 2007) and maize (Chatterjee et al., 2014). The strong expression of *AtBOR1* improved growth in both *Arabidopsis* (Miwa et al., 2006) and tomato (Uraguchi et al., 2014) under B-deficiency conditions. These results suggest that the greater accumulation of B eﬄux transporter could increase tolerance to B starvation through an enhanced B xylem translocation process. This is further supported by grape *VvBOR1* overexpression restoring the wild-type phenotype in an *Arabidopsis bor1-3* mutant (Pérez-Castro et al., 2012) and citrus *CmBOR1* overexpression increasing the tolerance to B deficiency in *Arabidopsis* (Cañon et al., 2013). Moreover, we recently found that ten aquaporin genes of roots were up-regulated in B-efficient Carrizo citrange, but only two of them were up-regulated in B-inefficient trifoliate orange at 24 h after B-deficiency treatment (Zhou et al., 2015). This result supports the proposal that the B-dependent regulation of aquaporins could affect the water status of the whole plant (Wimmer and Eichert, 2013). The plant aquaporin family functions in membrane channels and can facilitate the transport of water and other low molecular weight substances in the xylem (Ishibashi et al., 2011). Therefore, under B-deficiency conditions, Carrizo citrange may have a greater ability to absorb B and water and to transport them via the xylem to the shoot than trifoliate orange, thus tolerating B deficiency.

In contrast to the xylem, B retranslocation in the phloem can be achieved by two pathways: (i) xylem-to-phloem transfer along the stem, and (ii) retranslocation from the leaves (source) to the flowers or fruits (sink). Xylem-to-phloem transfer along the stem is particularly important for the B nutrition of plants during reproductive stage, due to the higher B demand and the lower transpiration rate of reproductive tissues than vegetative organs (Shelp et al., 1998; Huang et al., 2001). Moreover, B efficiency varies with plant growth period. For example, the wheat cultivar ‘Fang 60’ was B-efficient during reproductive growth, but vegetatively B-inefficient; the cultivar ‘SW41’ had the opposite trend of B efficiency compared with ‘Fang 60’ (Rerkasem and Jamjod, 1997). These results suggest that B efficiency of the plants at reproductive stage may be associated with the capacity for xylem-to-phloem transfer along the stem. The other pathway is the B retranslocation from the leaves to the flowers or fruits. After arriving at the source leaves, B can be retranslocated into the sinks (e.g., flowers, fruits) via the phloem by forming complexes with hydroxyl groups (the main pathway of B retranslocation; Brown and Shelp, 1997); otherwise, B will leak back into the xylem due to its high membrane permeability (Oertli and Richardson, 1970). Boron retranslocation is more marked at low B supply than at adequate or high B supply (Liakopoulos et al., 2009). Significant ^10^B retranslocation was found in the new stem and needles of Scots pine and Norway spruce seedlings after applying ^10^B to old needles, presumably as a consequence of B forming complexes with pinitol or inositol (Lehto et al., 2004a). In the olive tree leaf, mannitol may be involved in the promotion of B retranslocation under B limitation (Liakopoulos et al., 2009). Therefore, B retranslocation in the phloem is probably associated with the type and abundance of hydroxyl-bearing moieties, such as sugar alcohols (Perica et al., 2001a; Liakopoulos et al., 2009; Liu et al., 2014b). At the molecular level, B retranslocation may be associated with five B transporters (*AtBOR6*, *AtBOR7*, *OsBOR4*, *VvBOR1*, and *AtNIP6;1*) due to their expression in nodal regions of shoots or flowers (Tanaka et al., 2008; Fujiwara et al., 2010; Pérez-Castro et al., 2012; Tanaka et al., 2013). However, only *AtNIP6;1* and *OsBOR4* have been functionally identified (Tanaka et al., 2008, 2013). *AtNIP6;1* is highly expressed in nodal regions of shoots, particularly the phloem. The *nip6;1* mutants exhibit reduced expansion and low B concentrations of young rosette leaves under B-limited conditions (Tanaka et al., 2008). *OsBOR4* is specifically expressed in pollen, and *bor4* mutants show fertilization defect as a consequence of the reduced pollen tube elongation (Tanaka et al., 2013). These results suggest that *AtNIP6;1* and *OsBOR4* may function in B distribution into growing leaves or flowers. In addition, *AtNIP6;1* may be responsible for xylem-to-phloem transfer of B due to its predominant expression in the phloem of the nodes. Moreover, B retranslocation has further been partly revealed by the transgenic lines of sorbitol-related gene, *S6PDH*. This gene controls the activity of sorbitol-6-phosphate dehydrogenase, a key enzyme for sorbitol biosynthesis (Bellaloui et al., 2003). The overexpression of *S6PDH* could improve the retranslocation of B in plants by increasing sorbitol biosynthesis and thus enhance the tolerance to B deficiency in tobacco (*Nicotiana tabacum*; Brown et al., 1999) and rice (Bellaloui et al., 2003).

In trees, differences in B retranslocation efficiency may exist among genotypes within one species. Mattiello et al. (2009) found that in B-deficient eucalypt, the ratio of ^10^B: ^11^B in ^10^B-applied leaves decreased from 3.225 at day 1–1.492 at day 17 in clone 129 (*Eucalyptus grandis* ×*E. urophylla*), whereas that ratio only decreased from 2.759–1.900 in clone 68 (*E. grandis* ×*E. urophylla*). Accordingly, the ratio in other leaves remained stable at 0.245–0.254 in clone 68, whereas in clone 129, the ratio increased from 0.245 to 0.425. This result suggests that clone 129 is better able to retranslocate B to other parts after foliar application under B-deficiency conditions. Similarly, we observed that two orange scion cultivars with the same rootstock showed a different growth performance under B-deficiency conditions, likely due to the differences in B retranslocation efficiency (Sheng et al., 2009a). Although it is widely accepted that different degrees of B retranslocation among species are related to the level of polyols in the phloem (Brown and Shelp, 1997), little research on the B-polyol relationship among genotypes within a species has been conducted. Further studies are needed to examine the relationship between B retranslocation and polyols among genotypes within one species. Here, four approaches are recommended to assess the B retranslocation efficiency: (i) to evaluate B retranslocation by applying the stable isotope ^10^B in aerial plant parts (Brown et al., 1992; Lehto et al., 2004a; Leite et al., 2007); (ii) to identify the types of hydroxyl groups in leaf tissue and the phloem sap (Liakopoulos et al., 2009); (iii) to isolate and characterize the B-polyol complexes from leaf tissue and phloem sap (Hu et al., 1997; Penn et al., 1997; Stangoulis et al., 2010); and (iv) to compare B retranslocation between polyol-related trangenic mutant and wild type (Brown et al., 1999; Bellaloui et al., 2003).

### B Utilization

After long-distance transport, B enters into the targeted tissue, where it is required for the construction of the cell wall (O’Neill et al., 2001) and membranes (Voxeur and Fry, 2014) as well as for the metabolism of the cytoplasm (Brown et al., 2002). This process is known as B utilization. Boron use efficiency is related not only to the pectin content and composition of the cell wall (Hu et al., 1996; Kakegawa et al., 2005; Pan et al., 2012; Liu et al., 2013a), but also the cell membrane composition (Dordas and Brown, 2000). In general, B-efficient tree cultivars have a lower concentration of cell wall B at B-adequate supply but similar or greater concentration of cell wall B at B-limited supply, as compared to B-inefficient cultivars (Liu et al., 2013a; Wang et al., 2014). That is, B-efficient cultivars may possess a higher proportion of non-cell wall B which is available for cytoplasmic metabolism under B-adequate conditions, but a greater proportion of cell wall B which is required for the structure of cell wall under B-limited conditions (Hu et al., 1996; Pan et al., 2012). Moreover, the pectin composition of B-efficient cultivars differs from that of B-inefficient cultivars. For example, B-efficient Carrizo citrange had a higher concentration of CDTA-soluble pectin than B-inefficient trifoliate orange under B-limited conditions (Liu et al., 2013a). Boron use efficiency may also be associated with the cell membrane composition. The *Arabidopsis chs1-1* mutant, which has a lower proportion of sterols than the wild type, showed a 30% higher B uptake, whereas the *act1-1* mutant, which has an increased percentage of longer fatty acids, exhibited a 35% lower B uptake than the wild type (Dordas and Brown, 2000). At the molecular level, Miwa et al. (2013) demonstrated that the proportion of cross-linked RG-II in *Arabidopsis bor2-1* and *bor2-2* mutants (∼42.5 and ∼45.7%) was significantly lower than that of both wild type (∼54.0%) and *bor1-3* mutant (∼52.8%) plants under B limitation. These results may suggest that B transport by the *At*BOR2 protein from the symplast to the apoplast may be required for the effective cross linking of RG-II in the cell wall under B deficiency. Transgenic lines with enhanced expression of BOR2 showed improved root growth and better fertility under low B conditions, suggesting the potential utility of BOR2 expression in agricultural applications (Takada et al., 2014).

### Future Prospects

In summary, at a physiological level, B efficiency in trees is mainly attributed to four mechanisms: (i) the ability to absorb B from the soil/medium, which depends on root morphology and mycorrhiza; (ii) B translocation from root to shoot as indicated by the composition of root cell sap and xylem exudate and likely influenced by xylem vessel characteristics and water use efficiency; (iii) B retranslocation through xylem-to-phloem transfer and formation of complexes with hydroxyl groups in phloem; and (iv) the B requirement in cell wall construction and cell membrane composition. At a molecular level, the tolerance to B deficiency can be improved by the higher or stronger expression of *NIPs*, *BORs*, and *S6PDH* to facilitate B uptake, B translocation and retranslocation, as well as B utilization processes. These molecular results indicate that genetically modified trees with B-deficiency-tolerance-related genes may be useful in forestry or other tree industries in the future. Furthermore, the *bor1 bor2* double mutants exhibited more severe growth defects under B-limited conditions than *bor1* or *bor2* single mutants in *Arabidopsis* (Miwa et al., 2013), indicating that B-deficiency-tolerance-related genes are probably dosage-dependent. That is, the differences in B efficiency probably originate from a combined effect of the four mechanisms mentioned above. However, previous studies were mainly limited to one single mechanism of B efficiency. Consequently, more systematic study is needed on the B deficiency tolerance mechanisms in trees, including uptake, translocation, retranslocation, and utilization, spanning investigations from the physiological to the molecular level.

## Concluding Remarks

Boron deficiency is frequently observed in woody plants. An adequate B supply for cultivated trees can be of great economic importance, contributing significantly to the yield and quality of fruit and forest trees. From a practical view point, there is a need to focus research on the significance of B reserves, which is especially important in trees, for which there may be no early warning symptoms of B deficiency. Species or genotypes with different B efficiency have provided ideal experimental material to elucidate some puzzling aspects of the mechanisms for tolerance to B deficiency. Recently, great progress has been achieved in uncovering the molecular mechanisms of B efficiency in herbaceous plants, which makes possible further exploration of the mechanisms of B deficiency tolerance in woody plants.

## Conflict of Interest Statement

The authors declare that theresearch was conducted in the absence of any commercial or financial relationships that could be construed as a potential conflict of interest.
